# Unique underlying principles shaping copper homeostasis networks

**DOI:** 10.1007/s00775-022-01947-2

**Published:** 2022-07-08

**Authors:** Lorena Novoa-Aponte, José M. Argüello

**Affiliations:** 1 Department of Chemistry and Biochemistry, Worcester Polytechnic Institute, 60 Prescott St, Worcester, MA 01605 USA; 2 Genetics and Metabolism Section, National Institute of Diabetes and Digestive and Kidney Diseases, NIH, Bethesda, MD 20892 USA

**Keywords:** Copper, Bacteria, Homeostasis, Modeling, Systems biology

## Abstract

**Abstract:**

Copper is essential in cells as a cofactor for key redox enzymes. Bacteria have acquired molecular components that sense, uptake, distribute, and expel copper ensuring that cuproenzymes are metallated and steady-state metal levels are maintained. Toward preventing deleterious reactions, proteins bind copper ions with high affinities and transfer the metal via ligand exchange, warranting that copper ions are always complexed. Consequently, the directional copper distribution within cell compartments and across cell membranes requires specific dynamic interactions and metal exchange between cognate *holo-apo* protein partners. These metal exchange reactions are determined by thermodynamic and kinetics parameters and influenced by mass action. Then, copper distribution can be conceptualized as a molecular system of singular interacting elements that maintain a physiological copper homeostasis. This review focuses on the impact of copper high-affinity binding and exchange reactions on the homeostatic mechanisms, the conceptual models to describe the cell as a homeostatic system, the various molecule functions that contribute to copper homeostasis, and the alternative system architectures responsible for copper homeostasis in model bacteria.

**Graphical Abstract:**

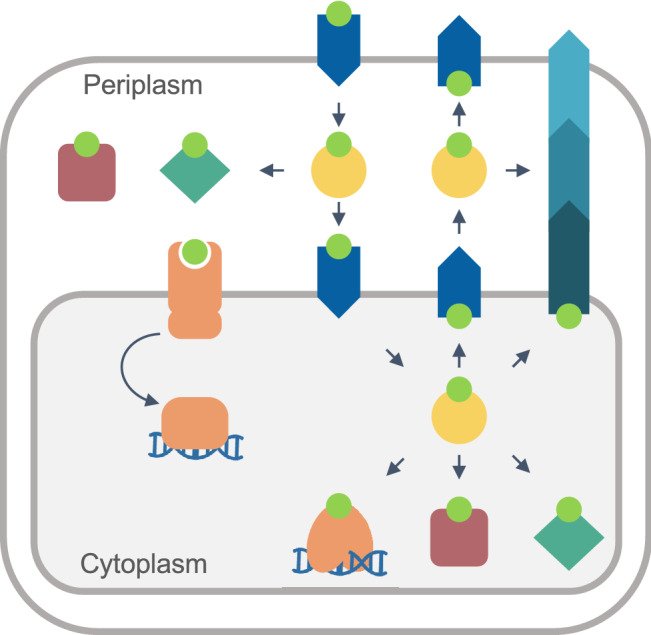

## Introduction

The emergence and evolution of copper distribution, sensing, and eventual storage mechanisms have been driven by the physiological role of the metal; this is, its participation in redox reactions as a prosthetic group of metalloenzymes [1–6]. In this context, copper homeostasis could be described as that of any other non-metabolizable nutrient requiring tunable input/output mechanisms. However, the reactivity of copper ions with diverse biomolecules generates the need for high-affinity copper binding molecules ensuring the absence of free (hydrated) copper in the cellular milieu [7–9]. This is, the homeostasis of copper in biological systems requires intra-compartmental copper binding chaperones, transmembrane unidirectional transporters, and metal level sensing transcriptional regulators. These bind the metal with high affinity and engage in the practically irreversible metal transfer among cognate molecules [10–12]. We center this review on bacterial studies, as copper homeostasis is arguably better understood in these organisms. We will refer to Cu^+^, rather than undefined copper, as this oxidation state is the substrate of the various homoeostatic proteins [13, 14]. Nevertheless, Cu^2+^ will be referred to when its expected involvement in enzymatic and non-enzymatic reactions is addressed.

The deleterious reactions of free Cu^2+/+^ were the driving force for early studies focusing on assessing bacterial tolerance to external Cu^2+^ by measuring growth rates and live/death phenotypes [15, 16]. These supported the involvement of active transporters (CopA, CusCBA) in cytoplasmic Cu^+^ efflux, small chaperoning proteins (CopZ, GolB) that carry Cu^+^ to punctual targets, and Cu^+^ sensing transcriptional regulators (CueR, CopY, CsoR) [4, 17–19]. The limited information on bacterial cuproenzymes and their metallation, as well as an incomplete grasp of membrane impermeability and transporter substrate selectivity, also contributed to descriptions of Cu^+^ homeostasis centered on cytoplasmic Cu^+^ sensing regulators and efflux mechanisms. Moreover, Cu^+^ distribution ideas have been primarily based on affinity constants of aqueous metal binding to these various molecules [20, 21].

In this review, we aim to provide an integrated view of dynamic Cu^+^ homeostasis networks operating in model bacteria under steady-state conditions. First, we will consider the properties of Cu^+^ sites in the proteins forming the core system of Cu^+^ mobilization/usage, as well as the kinetics and thermodynamic characteristics of Cu^+^ transfer. Second, we will evaluate the dynamic integration of protein expression and transport rates toward mathematically describing the steady-state Cu^+^ levels in a temporal space compatible with fast dividing bacteria. Third, we will review the various sensing, chaperoning, and transporting molecules in the context of cellular compartments delimited by Cu^+^ impermeable membranes. Finally, we will discuss the alternative molecular architectures/strategies to achieve Cu^+^ homeostasis present in different model bacterial species.

## Transient high-affinity Cu^+^ binding: a determinant element of the homeostatic system

An analysis of the structure–function of Cu^2+/+^ binding sites in proteins shows a clear difference among those found in cuproenzymes, and those in sensors, chaperones, and transport proteins responsible for metal cellular distribution [14, 22]. Enzymatic Cu^2+^ sites are classified as single electron transfer (type 1), mononuclear (type 2), dinuclear (type 3), or trinuclear (type 2 and type 3). These have distorted tetrahedral, planar square, and pyramidal coordination geometries with a preponderance of His residues, together with Cys and Met, as coordinating ligands. Importantly, cuproenzymes are metallated during the enzyme biogenesis and the ion remains bound throughout the functional life of the protein. There is no evidence that these sites participate in exchange reactions associated with Cu^+^ homeostasis nor do cuproenzymes appear to constitute sizeable metal pools. However, metal binding sites in Cu^+^ sensing and chaperone proteins are located on the protein surface and formed by two Cys residues in a quasilinear coordination. The structural biology of Cu trafficking has been the focus of excellent reviews by Rosenzweig and her group [14]. The structure of these binding sites aligns with the dynamic Cu^+^ exchange reactions involving equilibriums among their *apo* and *holo* forms [23, 24]. Similarly, Cu^+^ transporters only transiently bind the metal during transmembrane permeation. Transporters first exchange their substrate with soluble cognate partners via surface exposed sites. Cu^+^ binding sites involved in transmembrane translocation are located deep into the protein structures as shown by the structures of the isolated metal bound intermediates of Cu^+^ ATPases (CopA), tripartite antiporters (CusCBA), or the outer membrane porins (OprC) [25–27]. Nonetheless, they translocate the ion across the membrane and deliver to soluble partners in seconds.

Cu^+^ homeostatic proteins bind Cu^+^ with affinities in the fM-aM range (Table 1). Determinations of the high-affinity binding by transcriptional regulators controlling the expression of Cu^+^ chaperones and efflux transporters resulted in postulating the absence of free (hydrated) Cu^+^ in the bacterial cytoplasm [7]. This idea is based on that in a bacterium volume of 0.7–1.5 µm^3^, where fM-aM affinities can be formally satisfied by less than a single free atom in the system. However, equally relevant is the high affinity of all other homeostatic molecules (Table 1). Consider that to maintain all compartmental Cu^+^ bound, chaperones and possible metal sinking molecules should have similar fM-aM affinities as it would be quite inefficient, if not impossible, to compensate lower affinities by mass action [8, 9]. Similarly, high-affinity binding to transporters is required for forward transmembrane movement without backward leakage of free metal after receiving the ion from the chaperone [28–30].

**Table 1:** Cu^+^ binding affinities of representative members of Cu^+^ homeostasis networks in bacteria

Compartment	Molecule type	Name	Cu binding affinity (K_D_)	Organism	References
Cytoplasm	Cu-sensors/ transcriptional regulators	CueR	10^–21.0^ M	*Escherichia coli*	[204]
		CueR	10^–15.6^ M	*Pseudomonas aeruginosa*	[24]
		CopY	10^–16.6^ M	*Streptococcus pneumoniae*	[109]
		CsoR	10^–18.0^ M	*Mycobacterium tuberculosis*	[205]
		CsoR	10^–18.1^ M	*Staphylococcus aureus*	[100]
	Cu-chaperones	CopZ	~ 10^–18^ M	*Bacillus subtilis*	[206]
		CopZ	10^–14.8^ M	*Archaeoglobus fulgidus*	[10]
		CopZ1	10^–14.5^ M	*Pseudomonas aeruginosa*	[40]
		CopZ2	10^–16.1^ M	*Pseudomonas aeruginosa*	[40]
		CupA site 1	10^–17.9^ M	*Streptococcus pneumoniae*	[123]
		CupA site 2	10^–14.8^ M		
	Cu-exporters	CopA TM-MBS1	10^–15.1^ M	*Archaeoglobus fulgidus*	[27]
		CopA TM-MBS2	10^–15.0^ M		
		CopA N-MBD	10^–11.8^ M	*Archaeoglobus fulgidus*	[10]
Periplasm	Two component Cu-sensors	CopS*	10^–13.6^ M	*Pseudomonas aeruginosa*	[8]
	Cu-chaperones	CusF	10^–14.2^ M	*Escherichia coli*	[207]
		CopC*	10^–12.2^ M	*Pseudomonas syringae*	[9]
		PcoC*	10^–12.7^ M	*Escherichia coli*	[9]
		CopK*	10^–12.7^ M	*Cupriavidus metallidurans*	[208]

Similar values have been observed in homologous proteins from various species

^*^These proteins bind both Cu^+^ and Cu^2+^ with similar affinities

**Fig. 1:**
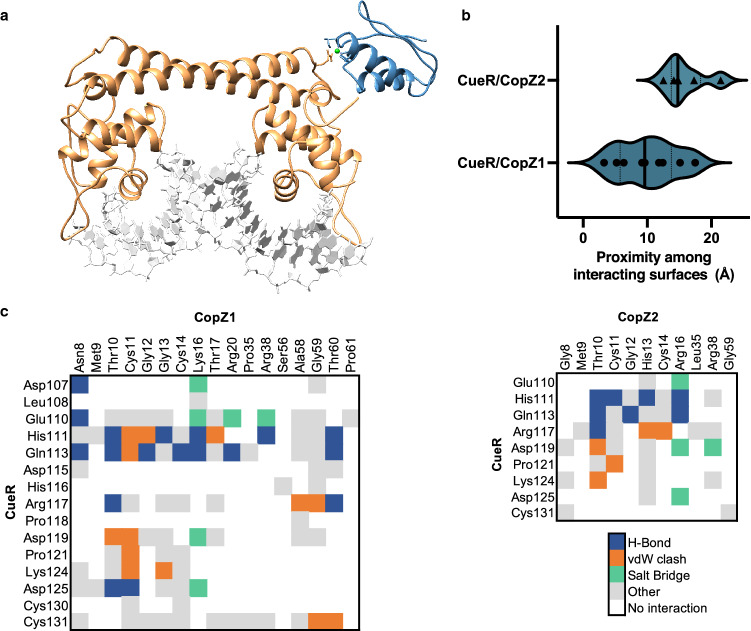
Docking of two homologous Cu^+^ chaperones with a Cu^+^ sensor. The *Pseudomonas aeruginosa* CopZ-like chaperones CopZ1 and CopZ2 were docked in silico onto the CueR regulator, and the energetics and the proximity among interacting surfaces calculated. **a** CueR-CopZ1 interaction model (conformer with lower interacting score). CueR (orange), CopZ1 (blue), Cu^+^ (lime), DNA (gray). **b** Black triangles and dots represent the proximity cluster averages resulted from docking experiments. Conformations in clusters from the CueR/CopZ1 interaction exhibit increased proximity. CopZ1 was confirmed in vitro and in vivo as the interacting partner delivering Cu^+^ to CueR. **c** Protein–protein bonding matrices for CueR/CopZ1 (left) and CueR/CopZ2 (right) show that stronger interactions are formed between CopZ1 and CueR. H-bonds (blue), salt bridges (green), van der Waals clashes (orange), and other hydrophobic interactions (gray) [24]

Tight binding to various sensing and distribution proteins seems contrary to Cu^+^ mobilization for the assembly of various cuproenzymes or export to the extracellular milieu. This is solved by the specific exchange of Cu^+^ among partnering proteins. Early analysis of Cu^+^ transfer among proteins considered the equilibrium metal binding *K*_*D*_ as the driving force for a vectorial movement, i.e*.*, the protein with the lower affinity transfers the ion to the protein with the higher affinity. While this might describe some Cu^+^ transfer reactions, it does not explain the directional metal distribution and discrimination among possible metal receiving targets. Other energetic determinants should be considered. In vitro, measured *K*_*D*_*s* describe the tendency of Cu^+^ to be bound to the ligating protein rather than hydrated in solution. In vivo, Cu^+^ transfer is dependent on the binding energy of Cu^+^ to each protein within the water-free protein/protein interphase environment. In addition, the metal-dependent interaction among specific partners is determined by the energetics of the involved non-covalent bonds [24, 29, 30]. Both, the bonding energetics and the proximity among interacting surfaces show how the docking interactions contribute to confer specificity to the metal exchange observed in vitro and in vivo between two homologous chaperones with a Cu^+^ sensor (Fig. 1) [24]. Moreover, the metal dependency of the protein/protein recognition appears key for efficient system operation as the interaction of *apo* partners appears futile and, except in the case of some chaperones, interacting *apo* partners cannot be isolated [24, 29]. An additional determinant in Cu^+^ distribution is the kinetics of metal exchange among cognate and non-cognate partners. The metal exchange is unlikely a rate-limiting step in Cu^+^ trafficking. Processes with large activation energies (transition states) such as transmembrane transport and cuproprotein metallation are the likely determinants of any Cu^+^ distribution process kinetics. Experimental evidence shows that short incubation times are required to observe Cu^+^ exchange [10, 24, 28, 29, 31]. However, even though the exchange among non-cognate partners might be assumed thermodynamically possible if only *K*_*D*_*s* are considered, the rate of transfer is largely reduced by a poor Cu^+^-dependent protein/protein recognition [24].

The high-affinity binding to the various homeostatic proteins and the consequent absence of free Cu^+^ has further consequences for the regulation of transport mechanisms. Consider the regulation of enzymatic/transport activities by free substrates as occurs in a glycolysis enzyme or the Na^+^/K^+^ ATPase. These enzymes are indeed regulated via transcription, chemical modification (phosphorylation), and targeting to membranes. Nevertheless, their primary regulation is by substrate concentration in a classical Michaelian fashion [32, 33]. When Cu^+^ is the substrate of transmembrane transporters, a Michaelian behavior is not possible since there is no free transported substrate in the cells. This is, although a Michaelian activation has been shown in vitro for both the free ion and the Cu^+^ chaperone, this cannot be achieved in vivo [10]. This explains the prevailing importance of transcriptional control in bacteria and membrane targeting in eukaryotes to regulate Cu^+^ transmembrane transport rates [34–36]. In other words, the rates of Cu^+^ efflux and uptake are not directly controlled by the substrate abundance but by the number of membrane transporters working at, or close to, *V*_max_.

## Progress toward system descriptions of Cu^+^ homeostasis

An approach toward understanding Cu^+^ homeostasis mechanisms is to ideate parsimonious model systems that can address biological and physicochemical constraints using various molecular elements (transporters, chaperones, storage pools, and sensors). It is relevant to go beyond only considering known participating molecules and propose elements that address functional requirements although the specific coding gene/functional protein might yet appear undiscovered. Such a system should be validated by mathematical models that, rooted in functional parameters (*V*_max_, *K*_*m*_, *K*_eq_*,* etc.), can describe experimental data. Then, the value of this exercise is in the capability of generating models that predict necessary elements and anticipate the consequences of experimentally altering the architecture of the system, increasing or decreasing the levels of participating molecules (i.e*.*, mutating or overexpressing a given gene).

**Fig. 2:**
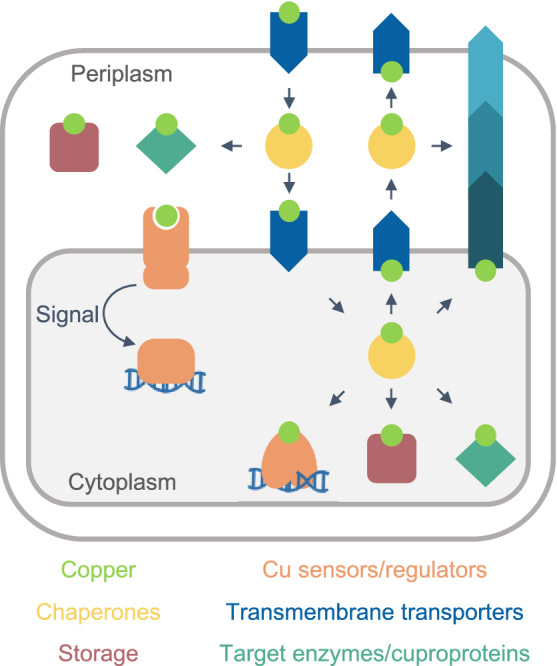
Conceptual minimum model of a Cu^+^ homeostasis network in a Gram-negative cell. Cu^+^ (lime), sensors (orange), storage sinks (rose squares), chaperones (yellow circles), transporters (blue arrows), and cuproproteins (turquoise diamonds). The metal is transported across the inner and outer hydrophobic membranes via specialized membrane-embedded transporters. The arrows indicate the direction of metal transport and vectorial transfer. Cu^+^ levels are sensed by a single-component (cytoplasm) or a two-component system (periplasm) that control the expression of all the elements of the Cu^+^ homeostasis network

A minimum model of a Cu^+^ homeostasis network in a Gram-negative organism is shown in Fig. 2. The existence of two hydrophobic barriers (inner and outer membranes) and two intracellular spaces (cytoplasm and periplasm) implies the presence of an interconnected network mobilizing Cu^+^ in each compartment, including sensors, storage, and chaperone proteins. Analogously, each membrane must include a set of importers and exporters. This conceptual model assumes that the driving force for the appearance of homeostatic mechanisms is the need to metallate cuproproteins, rather than to confer tolerance to toxic Cu^+^ levels. The engagement of free Cu^2+/+^ in Fenton reactions and the metallation of adventitious sites is undisputable. However, exposure to deadly Cu^2+^ levels is a rather rare event in nature; except perhaps during infection [37, 38]. More common appears the adaptation to moderate Cu^2+^ levels via efflux and binding to “sink” molecules [24, 39]. Indeed, we have observed normal growth rates in *Pseudomonas aeruginosa* [40] and *Salmonella enterica* (unpublished results) with intracellular steady-state Cu^+^ levels up to five times higher than those measured in unchallenged bacteria. This steady-state requires compartmental (periplasmic and cytoplasmic) sensing and storage mechanisms (Fig. 2), although these might yet to be discovered or functionally characterized in some organisms. In this context, the well-characterized Cu^+^ efflux ATPases, and other Cu^+^ efflux transporters, appear only as one of the elements required to maintain the steady-state rather than to survive under extreme conditions.

It is now known that metallation of membrane and periplasmic cuproproteins requires cytoplasmic Cu^+^ (see below) [41–43]. Still, cell membranes are impermeable to ions. This is an accepted principle of cell physiology [44, 45]. Then, membrane transporters mediating metal influx are necessary. We should also consider that ion transporters have exquisite selectivity mechanisms. For instance, the selectivity of K^+^ over Na^+^ in K^+^-channels is > 100 fold and > 300 fold in the outward-facing sites of the Na^+^/K^+^ ATPase [35, 46, 47]. In the case of transition metals, even larger selectivity can be expected when the competing substrates are bound to distinct chelating/chaperone molecules. In vitro we can tweak and test the ”promiscuity” of transition metal transporters [48–51]; however, in vivo conditions make permeation of Cu^2+/+^ by “piggybacking” or “leaking” through alternative transporters a very unlikely event. Furthermore, an influx through sporadic events would not justify the evolution and universal distribution of specific Cu^+^ sensors and efflux transporters, sustaining the metallation of essential cuproenzymes (cytochrome *c* oxidase, SOD), nor explain the experimentally determined fast Cu^+^ uptake [40, 52]. Consequently, the presence of specific Cu^+^ influx transporters in the outer and plasma membranes is predictable in every bacterial system (Fig. 2).

The presented minimal model does not discriminate likely parallel metal distribution pathways (Fig. 2). Consider, the side by side Cu^+^ efflux mediated by Cu^+^ ATPases and CusCBA-like transporters [4, 25], or that Cu^+^ pools feeding two alternative distribution pathways have been described [41–43, 52]. Similarly, Fig. 2 does not describe the different Cu^+^ distribution network architectures present in various bacteria. These provide distinct but equivalent solutions to similar biological and physicochemical constraints. These different strategies are frequently based on apparently “redundant” genes/proteins. However, the use of homologous proteins for metal targeting through alternative pathways is solved via specific protein/protein interactions directing the Cu^+^ exchange [24, 42, 53] (see below).

**Fig. 3:**
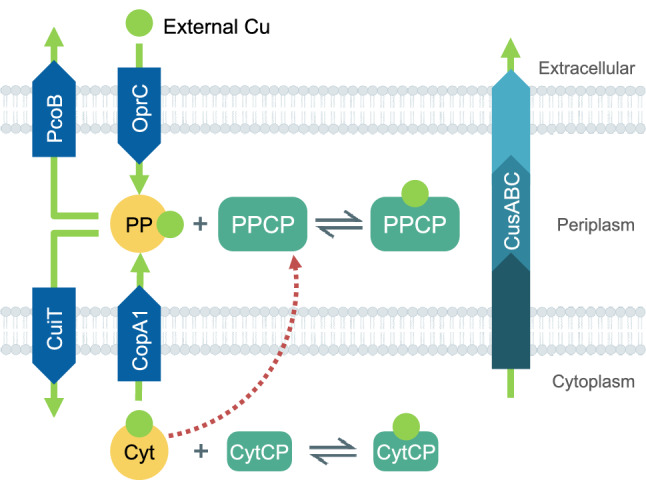
Compartmental distribution of Cu^+^ in a Gram-negative bacterium. The indicated transport and transfer reactions were included in a mathematical model of Cu^+^ uptake kinetics in *P. aeruginosa*. The model contemplates the change in compartmental Cu^+^ pools, the existence of parallel Cu^+^ efflux systems, the preponderance of a periplasmic Cu^+^ storage pool, and the transcriptional control of periplasmic storage proteins by cytoplasmic regulators (dotted red arrow). Metalloproteins exist in an equilibrium between their *apo* and *holo* forms. PP (periplasmic chaperones), Cyt (cytoplasmic chaperones). PPCP (periplasmic Cu^+^-binding proteins), CytCP (cytoplasmic Cu^+^ binding proteins). Green arrows represent the direction of Cu^+^ transport. The list of equations is detailed in the reporting manuscript [52]

The value of a model resides on its capability to describe a system via a mathematical representation that can be experimentally tested. Several models have been proposed to explain aspects of bacterial Cu^+^ homeostasis [52, 54–56]. Changes in Cu^+^ and in the chaperone transcripts levels upon Cu^+^ exposure have been modeled [54]. Cu^+^ binding affinities of transport ATPases, and the expression and degradation rates (at the transcription level) of regulators, chaperones, and efflux transporters were used as variables in the fitting equations. The obtained model performed well at predicting normalized steady-state levels of transcripts and intracellular Cu^+^ status after long exposure of *Halobacterium salinarum* (wild type and mutant strains lacking transporters and chaperones) in response to relatively low Cu^+^ levels. In other words, the model appears to predict the relative effects produced by the various mutations rather than the kinetics of the system response to Cu^+^ stress. In a different approach, we have modeled the kinetics of Cu^+^ influx in *P. aeruginosa* [52]. Based on the transport processes and metal transfer equilibriums, sets of simple chemical equilibrium and Michaelian equations were designed to describe the system (Fig. 3). This description could predict the Cu^+^ uptake kinetics in a range of external Cu^2+^ levels, the periplasmic and cytoplasmic Cu^+^ distribution, and simulate the dyshomeostasis generated by mutation of an involved transporter. Obtained parameters fairly represented those that have been experimentally determined, such as Cu^+^ ATPases transport rates and apparent *K*_*m*_s, and chaperone Cu^+^ affinities. Furthermore, developing the model confirmed various aspects of *P. aeruginosa* homeostasis mechanisms, including the transport of cytoplasmic Cu^+^ to the extracellular milieu by the CusCBA transporter, and the existence of Cu^+^ influx transporters. While the mathematical modeling provided significant insights, some weaknesses were evident. For example, it is well established that the expression of efflux transporters and chaperones increases upon exposure to external Cu^2+^. However, our model only considered an increase in the expression of a periplasmic storage pool. The independence of the model on the expression of additional genes is driven by the > 90% confidence achieved in the simulated reactions. In other words, the improvement in the fitting by adding further elements would be indiscernible [52]. Further refinement of a computational approach to describe the Cu^+^ homeostatic components might require monitoring other functional characteristics as for example the *holo/apo* ratios of nodal proteins.

## Molecules involved in Cu^+^ homeostasis

Further progress in our understanding of bacterial Cu^+^ homeostatic mechanisms might derive from the analysis of the molecules supporting the various functional components (Figs. 2 and 3).

### Cuproenzymes

Numerous cuproenzymes are present in bacteria [57, 58]. These include cytochrome oxidases *cbb*_*3*_, *aa*_*3*_, *caa*_*3*_ [1, 59], nitrite reductase, nitric and nitrous oxidoreductases [60, 61], Cu/Zn-superoxide dismutases [62–64], plastocyanin and azurin [65, 66], the small blue multicopper oxidases (MCO), laccases [5, 67, 68], NADH dehydrogenase-2 [69, 70], tyrosinases [6, 71], methane monooxygenases, amine oxidases, and polysaccharide oxygenases [67, 72]. Our goal is to point out characteristics of their metallation that influence Cu^+^ distribution/homeostasis; in particular, Cu^+^ chaperones, sensors, and transport across the plasma membrane. As mentioned, different from the Cu^+^ binding to chaperones, transporters, and sensors, catalytic Cu^2+/+^ remains bound during the functional life of cuproenzymes. These permanent interactions involve higher coordination numbers and more complex geometries [14, 50]. Metallation of enzymes with accessible sites might be mediated post-translationally via chaperone delivery as proposed for bacterial *bcc*_*3*_ [73] or the eukaryotic SODs [62]. However, the limited accessibility of Cu^2+/+^ sites in the structures of many cuproenzymes implies that in some cases the metal must be incorporated during protein folding, or in the case of enzymes embedded in the membrane, following the polypeptide translocation. In any case, this is not an aleatory process and, consequently, cuproenzymes metallation should involve dedicated Cu^+^ chaperones and transporters. Most of the identified bacterial cuproenzymes are located either on the plasma membrane, at the periplasm, or on the cell surface. This absence of cuproenzymes residing in the cytoplasm may mislead us to assume that there is no need for a pool of cytoplasmic Cu^+^ available for protein metallation. However, the presence of plasma membrane Cu^+^ influx and efflux transporters dedicated to Cu^+^ mobilization specifically targeted for protein metallation has been shown [41–43]. Consideration of secreted and membrane proteins synthesis in bacteria provides clues on how cytoplasmic Cu^+^ is targeted for protein metallation. In bacteria, two main mechanisms are involved in protein transport across, or insertion into, the bacterial membrane: the general secretory (Sec), and the twin-arginine translocation (Tat) pathways [74, 75]. In the Tat pathway, soluble proteins are partially folded in the cytoplasm before translocation. Tat-targeted cuproproteins include bacterial MCOs, cuproenzymes from the bacterial denitrification pathway, and polysaccharide oxygenases [76–78]. In the denitrification pathway, the periplasmic metallation of NosZ appears mediated by the Cu^+^ binding chaperone NosL [79]. The *Escherichia coli* CueO, an MCO member of the cupredoxin family with four Cu ions bound at two sites (a mononuclear Cu center, and a trinuclear Cu cluster) is folded in the cytoplasm and translocated in its *apo* form to the periplasm through the Tat pathway [80, 81]. How CueO receives Cu^+^ remains to be determined. Recent studies of the *Rhodobacter capsulatus* MCO CutO have shown that the enzyme metallation requires a likely chaperone, the periplasmic Cu^+^ binding CutF, and the plasma membrane Cu^+^ ATPase CopA [82]. Interestingly, *R. capsulatus* Cu^+^ ATPase CcoI, while required for *bcc*_*3*_ metallation, does not distribute Cu^+^ for the metallation of CutO [82]. This points to parallel pathways used to metallate alternative cuproenzymes.

In the Sec pathway, proteins are secreted via co-translational translocation and acquire the metal cofactor in the periplasmic space. Examples of Sec secreted proteins include the simplest members of the cupredoxin superfamily, azurin, and plastocyanin. These possess Sec signal peptides and bind a single Cu^2+/+^ atom at one end of an antiparallel β-barrel structure [83]. The *cbb*_*3*_ cytochrome *c* oxidase is also inserted in the membrane via the translocon in the Sec pathway [84–86]. Dedicated cytoplasmic Cu^+^ importers (CcoA) and Cu^+^ exporters (CcoI), together with two periplasmic chaperones (PccA to SenC), form the relay system for directing the metal specifically to *cbb*_*3*_ [42, 73, 87]. These observations confirm the need for Cu^+^ channeling through the cytoplasm even for Sec secreted proteins. Another example of Sec-secreted periplasmic Cu^+^ enzymes are the soluble Cu/Zn-SOD (SODCI-II) from *S. enterica* [88, 89]. In this case, a plasma membrane Cu^+^ ATPase, either CopA or GolT, and the putative periplasmic chaperone CueP are necessary for SODC-II metallation [41]. While the evidence for cytoplasmic control of periplasmic metallation is abundant, these studies are largely based on the phenotypical analysis of mutant strains. The metal transfer from membrane ATPases to periplasmic chaperones and from these to target enzymes remains to be characterized.

### Cu^+^ sensors

Bacteria need to maintain a steady-state Cu^+^ quota, ensuring that cuproenzymes are metallated while responding to sudden changes in the intra/extracellular Cu^2+/+^ levels. This is mediated by transcriptional regulators that sense the bioavailability of Cu^+^ in the cell compartments and remodel the Cu^+^ homeostasis network landscape avoiding both metal deprivation and toxicity. Cytoplasmic Cu^+^ sensing is performed by one-component sensors (CueR, CsoR, and CopY), where a single protein binds Cu^+^ and regulates gene expression [35, 90, 91]. In contrast, periplasmic Cu^2+/+^ sensing is mediated by membrane-associated two-component systems (TCSs) (CopR/S, CusR/S, PcoR/S). In these molecules, one component senses metal levels in the periplasm and transduces the signal to a cytoplasmic partner that exerts the transcriptional response to maintain the periplasmic Cu^+^ quota [35, 92].

Present in most proteobacteria, CueR (Cu Efflux Regulator) is a member of the MerR family of transcriptional regulators, usually controlling the expression of a Cu^+^ ATPase (CopA), a chaperone (CopZ), and a MCO (CueO) [35, 90]. CueR is a homodimer that binds tightly two Cu^+^ ions per protomer using Cys residues (Table 1). In the cell cytoplasm, where there is no free Cu^+^, the *holo* chaperones (CopZs) will transfer Cu^+^ to the *apo* sensor (Fig. 1). In other words, under physiological conditions, the sensor responds to the pool of Cu^+^-CopZ rather than free metal [24]. CueR transcriptional control occurs via a DNA distortion mechanism. Both *apo*-CueR and *holo*-CueR bind DNA with similar affinities at the promoter regions of regulated genes. Under non-activating conditions (i.e., low cytoplasmic Cu^+^-CopZ), *apo* CueR maintains the bound DNA in a conformation that prevents transcription by impeding the RNA polymerase interaction with the promoter region. Alternatively, *holo* CueR induces a kinked DNA conformation that allows the transcription to proceed [93–97]. The dissociation of the metal from the sensor to *turn off* the transcriptional activation seems unlikely given the Cu^+^ binding affinity of CueR. Instead, single-molecule studies have shown that a soluble pool of *apo*-CueR directly substitutes the DNA-bound *holo* CueR to terminate transcription [95]. In vivo analysis of the *apo/holo* CueR ratios under various conditions would provide interesting information on the process.

CsoR and CopY are cytoplasmic Cu^+^ sensing transcriptional repressors. CsoR is the founding member of a large class of sensing repressors responding to varied signals [98]. The CsoR homologs are found in various Gram-positive bacteria [99–104]. CsoR Cu^+^ sensors are homotetramers that bind Cu^+^ with high affinity (Table 1) via a trigonal S_2_N coordination geometry with two Cys, and a His residue [98, 100]. The *apo* CsoR binds DNA preventing gene expression. Cu^+^ binding to CsoR allosterically decreases the sensor affinity for DNA, freeing the promoter region with the consequent expression of *copA and copZ*, which mediate cytoplasmic Cu^+^ efflux [98, 100, 103]. In vitro evidence suggests that CsoR also obtains Cu^+^ from *holo* CopZ [103]. The CopY repressor, absent in eubacteria, was first described in the Gram-positive *E. hirae* as part of the *copYZAB* operon [105]. The Cu^+^ CopZ transfers two Cu^+^ ions to CopY causing its release from DNA and the concomitant de-repression of the CopY operon [106, 107]. Different from the Cu^+^ sensors described above, the homodimer CopY can also bind a Zn^2+^ at the Cu^+^ site [108, 109]. This imposes an alternative mechanism for the activation/deactivation of the CopY repressor functionality. Zn^2+^ appears as an allosteric activator of CopY DNA binding required for full repression of the *cop* operon in the absence of Cu^+^ stress [108–110]. This is, the *cop* operon is repressed by the Zn^2+^-bound CopY homodimer, once Cu^+^ levels rise, Cu^+^ displaces Zn^2+^ impairing CopY interaction with DNA leading to the de-repression of the regulated operon [109].

Three types of periplasmic Cu^2+/+^ sensing TCSs have been described in bacteria: the chromosomally encoded CusRS and CopRS, and the plasmid-borne PcoRS. Their molecular strategy responds to the need of sensing Cu^2+/+^ in one compartment (the periplasm) and performing the transcriptional control in another compartment (the cytoplasm). The TCS systems control the expression of putative outer membrane metal efflux transporters and periplasmic soluble proteins (chaperones, MCO, storage pools, and proteins of unknown function). For instance, *E. coli* CusRS controls the expression of the *cusCFBA* operon [92, 111, 112]; *E. coli* PcoSR controls two operons, *pcoABCD* and *pcoRS*, plus a separate *pcoE* gene [113–115]; and *P. aeruginosa* CopRS, regulates the expression of the operon *pcoAB*, along with the genes *ptrA*, *PA2807*, and *queF* [8, 40]. The sensor polytopic membrane proteins are homodimers with a Cu^2+/+^-binding region facing the periplasmic space and a cytoplasmic phosphotransferase domain [35, 92, 111]. The periplasmic sensors bind Cu^2+/+^ at the dimeric interphase using Met/His residues [8, 116]. Interestingly, the sensors bind both forms Cu^2+^ and Cu^+^ with similar high affinity (Table 1). This suggests the possibility that, under alternative physiological conditions, either Cu^2+^ and Cu^+^ might be present in the periplasm.

In the archetypical CusRS system, metal binding to CusS triggers the allosteric activation of the kinase activity in the phosphotransferase domain. This leads to phosphorylation of CusR and expression of the *cus* regulon. Deletion of either CusRS component leads to increased intracellular Cu^+^ due to the lack of expression of the *cus* regulon [92, 111, 112, 114, 116]. Alternatively, *P. aeruginosa* TCS CopRS relies on the metal-driven allosteric inhibition of the constitutive phosphatase activity of CopS, rather than on its kinase activity [8]. This is, *P. aeruginosa* CopR is constitutively phosphorylated by an undetermined unspecific kinase. In the absence of periplasmic Cu^2+/+^, CopS shuts down the transcriptional response by dephosphorylating CopR. Once Cu^2+/+^ binds CopS, its phosphatase activity is blocked and the phosphorylated CopR turns *on* the expression of the CopRS regulon. In consequence, *P. aeruginosa* strains lacking CopS are not sensitive to Cu^2+^ since the CopRS regulon is constitutively expressed [8]. This mechanism requires a crosstalk with an unknown kinase required for the phosphorylation of CopR. This kind of interactions have been also suggested for *E. coli* CusS, based on in vitro data showing that although CusS only phosphorylates CusR, the cytoplasmic regulator can be phosphorylated by non-cognate TCS kinases, including YedV, BarA, and UhpB [117].

It should also be noted that different aspects of Cu^+^ sensing and the regulation of homeostasis are still unclear. For instance, the regulation of Cu^2+/+^ influx transporters has not been established, even though their expression is downregulated upon Cu^+^ exposure. Alternatively, it is not known how the periplasmic sensors acquire/release metal or whether under in vivo conditions they can sense both Cu^2+/+^.

### Cu^+^ chaperones

Connecting metal uptake, sensing, storage, utilization, and elimination, Cu^+^ chaperones play a central role in the homeostatic network. They have two main functions: to ensure Cu^+^ delivery to *apo*-proteins where Cu^+^-dependent protein–protein recognition is determinant, and to prevent Cu^+^ from engaging in harmful redox chemistry acting as a high-affinity buffer system. The best characterized family of Cu^+^ chaperones is the cytoplasmic CopZ-like proteins present in most bacterial species [24, 40, 101, 107, 118–121]. These are small (~ 7 kD) soluble proteins with a ferredoxin fold, homologous to the regulatory domains present at the N-terminus of Cu^+^ ATPases (CopA) [14, 24, 122]. Like those regulatory domains, chaperones bind Cu^+^ with apparent *K*_*D*_*s* on the sub-fM range using accessible thiol side chains in an invariant CxxC motif (Table 1) [8, 9]. CopZs are encoded by single genes usually in an operon with a cognate Cu^+^ ATPase. However, *E. coli* CopZ is expressed from the gene encoding the CopA transporter, as a result of a ribosomal programmed frameshifting [120]. The archetypical role of CopZ proteins is the trafficking of Cu^+^ to the transport (transmembrane) and regulatory (cytoplasmic) sites of CopA, and to Cu^+^ sensing transcriptional regulators (CopY, CsoR, and CueR) [10, 24, 29, 103, 107, 123]. The Cu^+^ transfer is mediated by the protein/protein recognition that occurs between the chaperone and its cognate partners, allowing the ligand exchange without releasing the Cu^+^ into solution. This is evident in the Cu^+^ delivery by CopZ to CueR (Fig. 1), and the Cu^+^ ATPases transport mechanism. In the later, the electronegative surface of the *holo* chaperone docks with an electropositive platform in the ATPase structure. The docking of the *apo*-chaperone is unstable and after Cu^+^ delivery, the protein/protein complex disassembles [28]. It is significant that some bacterial species have more than one gene encoding CopZ-like proteins; for example, *P. aeruginosa* [40], *Streptomyces lividans* [103], or some Rhizobiales [53, 124]. Characterization of *P. aeruginosa* CopZ1 and CopZ2 has shown that, rather than redundant proteins, they constitute independent Cu^+^ pools. While the less abundant CopZ1 acts as the chaperone delivering Cu^+^ to the sensor CueR, CopZ2 functions as a fast response Cu^+^ sequestering storage protein. In line with this role, CopZ2 expression is strongly upregulated upon Cu^2+^ exposure [24].

Distinct from the soluble CopZ chaperones, the *S. pneumoniae* CupA is a plasma membrane-anchored Cu^+^ chaperone. Unlike the characteristic ferredoxin fold of CopZs, CupA adopts a cupredoxin-like structure with a binuclear Cu^+^ configuration where site S1 is a high-affinity Cu^+^ site and site S2, a Met-rich low-affinity binding site [110, 125]. The S1 Cu^+^ binding site is accessible, allowing CupA to deliver Cu^+^ to the regulatory N-terminal metal-binding domain (MBD) of the Cu^+^ transport ATPase CopA and to the transcriptional regulator CopY [110, 123, 125].

The Cu^+^ binding affinities of periplasmic sensors and established chaperones indicate the absence of free Cu^+^ in this compartment (Table 1). Then, it is predictable that periplasmic chaperones will link the bidirectional Cu^+^ trafficking between the outer and plasma membrane transporters and mediate the distribution of Cu^+^ for cuproenzymes metallation (Fig. 2). The periplasmic CusF chaperone, part of the CusCFBA efflux system, receives Cu^+^ from the plasma membrane ATPase CopA and delivers the metal to CusB that further translocate Cu^+^ to the extracellular milieu [28, 31, 126, 127]. *E. coli* CusCFBA expression is under the control of the two-component periplasmic Cu^+^ sensor CusRS, implying a role of CusF in metal efflux [128]. CusF is a beta-barrel protein with an exposed Met_2_His motif responsible for Cu^+^ binding [129–131]. Its binding affinity is comparable to that of periplasmic Cu^+^ sensors (Table 1) and supports the idea that, like in the cytoplasm, there is no free Cu^+^ in the periplasm [8, 132]. Moreover, the Cu^+^ transfer mechanism from the CopA ATPase to CusF further guarantees the absence of free Cu^+^ in the periplasm. In this process, the *apo* CusF docks with the exit of the Cu^+^ permeation path of the transporting ATPase, preventing the release of the free ion after transmembrane transport. The *holo* chaperone does not bind the ATPase and departs from the docking site carrying the ion to CusB [31].

Considering the chaperones involved in cuproenzyme assembly, *S. enterica* CueP participates in the metallation of the Cu/Zn-superoxide dismutases SodCI and SodCII [41, 133, 134]. CueP is also upregulated by high Cu^+^ levels via the concerted action of the cytoplasmic Cu^+^ sensor CueR and the periplasmic stress sensor CpxR/CpxA [34, 135]. CueP appears to receive Cu^+^ from both Cu^+^ ATPases, CopA and GolT, although no CueP-coupled outer membrane transporter has been identified [41, 136]. Most periplasmic Cu^+^ chaperones involved in the metallation of cuproenzymes are not under the control of Cu^+^ sensing transcriptional regulators. These include both, ScoI/SenC and PCu(A)C, which are present in many species and deliver Cu^+^ to cytochrome oxidases [73, 137–140]. Similarly, *P. stutzeri* NosL is a membrane-anchored Cu chaperone that participates in the metallation of the nitrous oxide reductase NosZ [141–143].

Finally, although widely distributed, less is known about the periplasmic Cu^+^ chaperones from the Pco/Cop systems. PcoC and its homolog CopC are predicted periplasmic Cu chaperones [2]. A small set of CopCs contain distinct Cu^+^ and Cu^2+^ binding sites, but most bind a single Cu^2+^ with high affinity (Table 1) [144–148]. Their physiological roles are, however, not fully understood. Interestingly, a recent publication describes YobA, a CopC homolog, found on the *E. coli* chromosome as part of the AZY operon. YobA binds Cu^2+^ with a *K*_*D*_ in the nanomolar range. Although the deletion of *yobA* does not affect Cu^2+^ tolerance or the antioxidant defenses of the bacteria, cells lacking *yobA* exhibit a strong impairment of the NADH oxidation activity, suggesting that YobA might contribute to the metallation of the periplasmic cuproenzyme NADH dehydrogenase II [149].

Certainly, significant advancements have been made to identify chaperones and characterize their function. However, several questions remain; for example, which chaperones connect Cu^+^ transport across the outer and inner membranes or deliver the metal to periplasmic Cu^2+/+^ sensors? How do chaperones transfer the metal to cuproenzymes?

### Cu^+^ transmembrane transporters

Reaching steady-state levels of cellular Cu^+^ requires both influx and efflux transporters. Just few Cu^+^ influx proteins have been identified in bacteria: the outer membrane OprC, and the inner-membrane transporters CcoA and CuiT. The secreted siderophores have been also shown to mediate bacterial Cu^2+^ uptake; however, their selectivity under non-stress conditions, contribution to the overall metal uptake, and the kinetics of transport have not been characterized [150–152]. Consequently, their relative importance under physiological steady-state conditions cannot be evaluated. OprC is a channel-forming TonB-dependent outer membrane porin that binds Cu^2+^ and is downregulated in response to high external Cu^2+^ [40, 153, 154]. *P. aeruginosa* overexpressing OprC has increased intracellular copper levels [26]. OprC appears highly specific for Cu^2+/+^ transport, binding Cu^2+^ in a tetrahedral geometry via an unusual CxxxM-HxM motif [26]. Its structure also has a distinctive Met track formed by eleven Met residues that might guide Cu^2+/+^ from the extracellular surface toward the single Cu^2+^ binding site at the bottom of the track [26]. OprC-like proteins are present in some Proteobacteria (*P. stutzeri*, *S. enterica*, *Klebsiella pneumoniae)* but absent in *E. coli* [26, 155]. Studies have also suggested that OprC is important for the virulence of *P*. *aeruginosa* and *Acinetobacter baumannii* [156, 157]. However, the transport characteristics of OprC (*K*_*m*_*/V*_max_) and the putative transfer to periplasmic Cu^2+/+^ chaperones have not been explored. Similarly, how *oprC* is transcriptionally regulated is not clear, although there is evidence suggesting that the CueR Cu^+^ sensor binds the *oprC* promoter [158].

Regarding metal influx across the plasma membrane, we have identified a putative Cu Influx Transporter (CuiT) that, along with the outer membrane OprC, is downregulated in response to external Cu^2+^ [40]. In agreement with this, ∆*cuiT P. aeruginosa* accumulates less Cu^+^ upon exposure to the metal (unpublished results). CuiT is a member of the Major Facilitator Superfamily (MFS) present in numerous bacterial species. Large multiple sequence alignments (> 1000 sequences) show the invariance of potential Cu coordinating residues (Met/His) in central transmembrane segments of CuiT-like proteins (unpublished data). Originally identified in *R. capsulatus,* CcoA is a Cu^2+/+^ influx transporter required for *cbb*_*3*_ assembly [43, 86, 87]. This further supports the role of cytoplasmic Cu^+^ in the metallation of periplasmic cuproenzymes. Unlike OprC and CuiT, the expression of *P. aeruginosa* CcoA is not regulated in response to external Cu^2+^ [40]. In *P. aeruginosa* and *R. capsulatus,* the occurrence of multiple Cu-importers (CcoA and CuiT) mirrors the presence of two Cu^+^ efflux ATPases in those species (CopA1 and CopA2/CcoI) [42]. Thus, it is tempting to hypothesize that these are non-redundant parallel systems, part of distinct and independent Cu^+^ distribution networks. CcoA and CopA2/CcoI mediate Cu^+^ traffic to *bcc*_*3*_, while CuiT and CopA1 control the overall level of cytoplasmic Cu^+^. However, how do the single or multiple cytoplasmic chaperones discriminate among the various Cu^+^ ATPases to distribute the metals? Furthermore, understanding the interplay and physiological roles of these proteins would require defining their Cu^+^ transport kinetic parameters [42].

Arguably the best-characterized aspect of Cu^+^ homeostasis is cytoplasmic Cu^+^ export. P_1B_-type ATPases (CopAs) pump the metal out of the cytoplasm at the expense of the hydrolysis of ATP [10, 27, 159–163]. In parallel, the tripartite RND-type CusCBA effluxes Cu^+^ by using a proton-motive force [25, 112, 164, 165]. There are high-resolution structures available for both, the ATPases [166] and the CusCBA Cu^+^ efflux system [167]. In agreement with a role exporting Cu^+^, their inactivation usually results in Cu^2+^ sensitivity, increased intracellular Cu^+^ levels, and impaired virulence due to the incapacity of the cells to efflux the excess of metal [19, 162, 168–170]. Cu^+^ ATPases are polytopic membrane proteins with transmembrane metal-binding sites (TM-MBS). These are formed by six invariant residues that bind two Cu^+^ ions with high affinity in a trigonal planar coordination geometry (Table 1) [10, 27, 171]. CopAs transport mechanism follows the Post-Albers catalytic cycle characteristic of P-type ATPases [10, 29, 163, 172]. However, the substrate does not reach these transporters in a free form. Cu^+^ is delivered by *holo* CopZ to the TM-MBS via specific protein–protein recognition. The interaction between CopZ and CopA occurs via an electropositive platform located at the cytosolic side of the second TM helix of CopA, where the Cu^+^-loaded chaperones dock while delivering the ions to be transported. Cu^+^ is transferred via ligand exchange to a transient Cu^+^ site formed by invariant Met, Glu, Asp residues at the entrance of the transporter permeation path [28, 166]. After metal delivery, *apo*-CopZ leaves the docking platform. This, and the high affinity of TM-MBS, makes the transmembrane transport functionally irreversible [10, 173]. Upon the delivery, Cu^+^ ions are channeled to the high-affinity TM-MBS where they remain occluded until ATP hydrolysis induces deocclusion and metal translocation across the permeability barrier [28, 173]. Similar to the Cu^+^ transfer from Cu^+^-CopZ to the ATPase, a periplasmic chaperone should receive the metal from the ATPase to ensure that no free metal is released into the periplasm. The transfer from CopA to the periplasmic chaperone CusF, part of the *cusCFBA* operon, has been characterized. The electropositive surface of *apo*-CusF docks onto a negatively charged periplasmic loop of the ATPase, positioning the chaperone to receive the metal [31]. This vectorial relay of Cu^+^ from the cytoplasmic chaperone to the periplasmic chaperone contributes to maintain the absence of free Cu^+^ inside the bacterial cells.

Bacterial Cu^+^ ATPases contain one or two cytoplasmic N-terminal metal-binding domains (N-MBD) with ferredoxin folds. These bind Cu^+^ with an affinity similar to chaperones (Table 1) [10, 14, 171, 174]. N-MBDs exchange Cu^+^ with CopZ (*K*_eq_ ~ 1). The unidirectional Cu^+^ transfer from the membrane-anchored chaperone CupA to the CopA N-MBDs has also been documented [123]. Importantly, N-MBDs are dispensable for Cu^+^ transfer to the TM-MBSs [10]. Instead, N-MBDs appear to “sense” cytoplasmic Cu^+^ and regulate the turnover of the transporter. The *apo* forms of the domains interact with the ATP binding domain, upon binding Cu^+^ the N-MBDs “release” the catalytic domain allowing a faster turnover when the cytoplasmic Cu^+^ level raises (i.e., increased ATP hydrolysis activity) [29, 173, 175]. Referred to as CopA1-like, most identified ATPases are central to maintain low cytoplasmic Cu^+^ levels (CopA, CtpV, or CtaA). Alternatively, CopA2-like proteins (FixI, CcoI, or CtpA) supply Cu^+^ for the metallation of periplasmic cuproproteins (MCO CueO, Cu/Zn-SOD, *aa*_*3*_-type Cox, and *cbb*_*3*_-type Cox) [41, 42, 176–178]. While the expression of the *copA1*-like genes is regulated by the cytoplasmic transcriptional regulators (CueR, CopY, or CsoR), the expression of the *copA2*-like genes is not [42]. Despite both enzymes catalyzing the efflux of cytoplasmic Cu^+^ into the periplasm, CopA1-like proteins exhibit considerably higher transport rates. This coincides with the deletion of *copA1*-like genes severely impairing bacterial growth in the presence of Cu^2+^, while mutation of *copA2* has no effect on Cu^+^ toxicity nor on intracellular Cu^+^ levels [42]. It is clear that these are non-redundant similar ATPases; nevertheless, the link of the cytoplasmic Cu^+^ pool with the alternative ATPases is still poorly defined. This is, it is known that cytoplasmic Cu^+^ chaperones deliver the metal to the ATPases. However, it is unclear how the CopZ/CopA partnership is coordinated in organisms with multiple CopZ-like chaperones and CopA-like transporters, or how organisms with a single chaperone or a single Cu^+^ ATPase fulfill both requirements, Cu-tolerance and cuproenzymes metallation.

The tripartite CusCBA Cu efflux system is part of the resistance-nodulation-cell division protein superfamily (RND), spanning from the inner to the outer membrane in Gram-negative bacteria. The protein structure has a three-way symmetry (i.e., C_3_B_6_A_3_) where the periplasmic adaptor protein CusB links the outer membrane pore CusC with the inner membrane protein CusA [112, 128, 164, 179, 180]. The three components are encoded in a single operon, accompanied by the periplasmic chaperone gene *cusF* in some species [112, 126, 128, 179, 181]. The antiporter CusA binds Cu^+^ using a Met triad, and couples the transport with the energy of the proton gradient [167]. The periplasmic component CusB, is important for the assembly of the protein complex and also binds Cu^+^ via a Met triad in a trigonal planar geometry [182]. The alignment of the CusA trimer and the inner surface of the channel formed by CusB creates a continuous path for the metal. In turn, a Met triad of CusB appears as the entrance site for Cu^+^ delivered by CusF [164, 180]. Then, the Cu^+^ transfer between CusF and CusB is mediated by the transient protein–protein interaction and ligand exchange involving Met residues from both proteins [183]. Lastly, the outer membrane CusC trimer interacts with the CusBA complex forming the funnel toward the extracellular milieu. Cu^+^-binding residues have not been identified in CusC that appears to channel the metal through a series of electronegative residues [165].

It was proposed that *E. coli* CusCBA exports periplasmic Cu^+^ to the extracellular space [126, 179, 184]. However, cytoplasmic Cu^+^ export has also been postulated based on the structure and functionality of isolated CusA [167]. The regulation of CusCBA expression might help in discerning the underlying transport mechanism. As mentioned, Cu^+^ transport in bacteria is mainly transcriptionally regulated by modifying the transporters’ abundance. Intriguingly, *cusCBA* expression is regulated by a TCS periplasmic Cu^+^ sensor in some species and by a cytoplasmic Cu^+^ sensor in others. For example, *E. coli cusCBAF* operon is controlled by the TCS CusRS [128] but the *P. aeruginosa cusBCA* operon is regulated by the cytoplasmic CueR [40]. This would suggest that the cytoplasm Cu^+^ is the *P. aeruginosa* CusCBA substrate, which agrees with our modeling of the Cu^+^ fluxes in *P. aeruginosa* [52]. Coincidently, *P. aeruginosa* lacks the periplasmic chaperone CusF [185]. A role of CusCBA transporting cytoplasmic Cu^+^ implies that other mechanisms might mediate metal export from the periplasm to the extracellular milieu. Interestingly, the *Pseudomonas* TCS CopSR regulates the porin PcoB, for which a putative role on Cu^+^ export across the outer membrane has been proposed [115, 186, 187]. In agreement with a putative role in Cu^+^ detoxification from the periplasm, increased levels of Cu^+^ promote the expression of *pcoB* in the Gram-negatives *P. aeruginosa*, *Caulobacter crescentus*, and *A. baumannii* [8, 40]. Furthermore, Δ*pcoB* cells accumulated increased levels of intracellular Cu^+^ [186, 187] and displayed a moderated Cu^2+^ sensitive phenotype [186, 187]. However, Cu^+^ transport selectivity and kinetic experiments need to be performed to confirm the role of PcoB as a Cu^+^ transporter.

### Cu^2+/+^ storage

We have shown that in *P. aeruginosa* transcription of Cu^+^ exporter CopA1 and Cu^+^ chaperones CopZ1 and CopZ2 peaks at 5 min after adding Cu^2+^ to the media [24]. However, the efflux/influx steady-state is reached after 10 min. This is the time required for the synthesis of efflux transporters [40, 52]. Therefore, cellular Cu^2+/+^ storage might be proposed as an immediate response to metal overload before the full transcriptional response can be mounted. Various cytoplasmic molecules including GSH, metallothionein, and Csp1-3, have been suggested as potential Cu^2+/+^ storage systems in bacteria [39, 188]. We recently added a CopZ chaperone to this list [24].

The involvement of low molecular weight thiol molecules as glutathione (GSH) in Cu buffering has been proposed [20, 189]. However, the transcription of GSH synthesis proteins is not upregulated in response to Cu^2+^ stress [40, 117]. Moreover, while GSH binds Cu^+^ with sub-femtomolar affinity, Cu^+^-GSH complexes promote redox stress via the formation of superoxide, making GSH an unlikely physiological Cu^+^ sink [190–192]. In fact, the higher Cu^+^ affinity of cytoplasmic chaperones and transcriptional regulators likely limits any in vivo Cu^+^ binding to GSH [190]. The participation of GSH in tolerance of high Cu^+^ levels, or more likely the associated redox stress, appears evident only in mutant stains where the physiological Cu^+^ homeostasis machinery has been disrupted [193].

Cu-binding metallothioneins in bacteria were first described in *Mycobacterium tuberculosis* (MymT) as part of a regulon for Cu^2+^ resistance [104]. MymT is a small Cys-rich cytoplasmic protein that binds and sequesters Cu^+^ [188]. Although the expression of MymT is induced not only by Cu^+^ but also by Cd^2+^, Zn^2+^, Co^2+^, Ni^2+^, redox stress, and acid pH, cells lacking MymT are only sensitive to Cu^2+^, but no to other metals [188].

First described in the methane-oxidizing bacteria *Methylosinus trichosporium* OB3b, Copper Storage Proteins (Csps) are widely distributed (*P. aeruginosa*, *Bacillus subtilis*, *Streptococcus pneumoniae*, *S. lividans*, *S. enterica*, etc.) [39, 194]. There are three paralogues, Csp1 and Csp2 with a Tat signal for periplasmic localization, and the cytoplasmic Csp3. These Cys-rich tetrameric structures bind in vitro up to 52 (Csp1) and 80 Cu^+^ ions (Csp3), with affinities in the 10^17^ M^−1^ range [39, 194]. However, *B. subtilis* Csp3 heterologously expressed in a Δ*copA E. coli* strain showed a limited capability to rescue the growth defect associated with Cu^2+^ exposure [194, 195]. Alternatively, deletion of the *S. lividans* Csp3 orthologue led to a defect in Cu^+^ tolerance [196]. Interestingly, Cu^+^ transfer from isolated *S. lividans* CopZ to Csp3 was observed [196]. While Csp proteins avidly bind Cu^+^ in vitro, their physiological role remains to be established. Consider that their selectivity or capability to bind other metals has not been reported and the regulation of their expression in response to external Cu^+^ is not confirmed. Whether periplasmic and cytoplasmic isoforms have similar roles or if they bind other metals remains to be determined.

We have recently described the involvement of the chaperone CopZ2 in cytoplasmic Cu^+^ storage [24]. Under resting conditions, there is a pool of *apo* CopZ2 acting as a surveillance mechanism. The fast metal binding to CopZ2 appears as an initial translational/transcriptional independent response to Cu^+^ excess. CopZ2 expression is subsequently induced upon Cu^+^ overload and the protein reaches levels that commensurate with the cytoplasmic Cu^+^ quota [24]. CopZ2 levels were maximal 5–10 min upon Cu^2+^ challenge and remained elevated under steady-state conditions, i.e., constant high intracellular Cu^+^ content [24, 40]. Deletion of CopZ2 abolishes the cytoplasmic capacity to chelate Cu^+^. Interestingly, rather than a Cu^+^ susceptibility phenotype, overexpression of the exporter CopA1 is observed in cells lacking CopZ2 [24]. Other likely candidates for Cu^+^ storage (metallothionein, Csp3, or glutathione biosynthesis genes) do not participate in the *P. aeruginosa* response to high Cu^+^ stress [40].

There is an increase in the periplasmic Cu^2+/+^ pool upon Cu^2+^ stress that logically precedes a cytoplasmic overload [52, 197]. However, almost nothing is known about the proteins involved in periplasmic Cu^+^ storage. Various periplasmic proteins are induced in response to Cu^2+^ stress [40]. Some are expected to serve as Cu^+^ chelators, for example, the periplasmic Csp1 and Csp2 (vide supra) [39, 194]. Alternatively, several MCO and proteins from the Pco operon have been proposed as having a Cu^+^ binding role, rather than just a function of redox enzymes. For instance, PcoE binds Cu^+^ with picomolar affinity and its expression is controlled by CusRS [198]. We could also speculate a role for chaperones as CueP buffering Cu^+^ in the periplasmic space as its levels are higher upon Cu^2+^ exposure [133]. However, in vivo quantification of other proteins and *holo*/*apo* ratios under resting and Cu^2+/+^ stress conditions remain to be determined. This information is essential to assign these proteins a role in metal storage.

## Bacteria have adopted alternative architectures fulfilling the central functions associated with Cu^+^ homeostasis

Models of Cu^+^ homeostasis networks were proposed to describe systems addressing biological and physicochemical constraints and recent insights in the interplay of involved proteins and their transcriptional regulation (Figs. 2 and 3). The goal was to highlight common solutions and unifying mechanisms involved in Cu^+^ distribution. In this process, we generalized and grouped distinct molecules within common functions. However, there are also niche-specific adaptations and alternative strategies to address physiological needs. Various molecular architectures, still based on similar principles, are observed in the networks of different bacterial species. In this section, we will compare the Cu^+^ homeostasis systems in five bacterial pathogens (*P. aeruginosa, S. enterica, E. coli*, *S. pneumoniae*, and *M. tuberculosis*), highlighting parallelisms and divergencies. An increased molecular diversity is expected when moving from the common elements required for Cu^+^ homeostasis maintenance to the diverse Cu^+^ utilization by different cuproenzymes. The presence of singular cuproenzymes depends on the microenvironmental niche and bacterial adaptations to available resources (*e.g.,* denitrification, biofilm formation, or intracellular pathogenesis). In any case, there is limited information on the range of Cu^+^ delivery systems dedicated to protein metallation, their regulation, and the mechanism of Cu^+^ insertion as part of protein maturation. For simplicity, we have not included proteins involved in cuproproteins metallation in these diagrams.

**Fig. 4:**
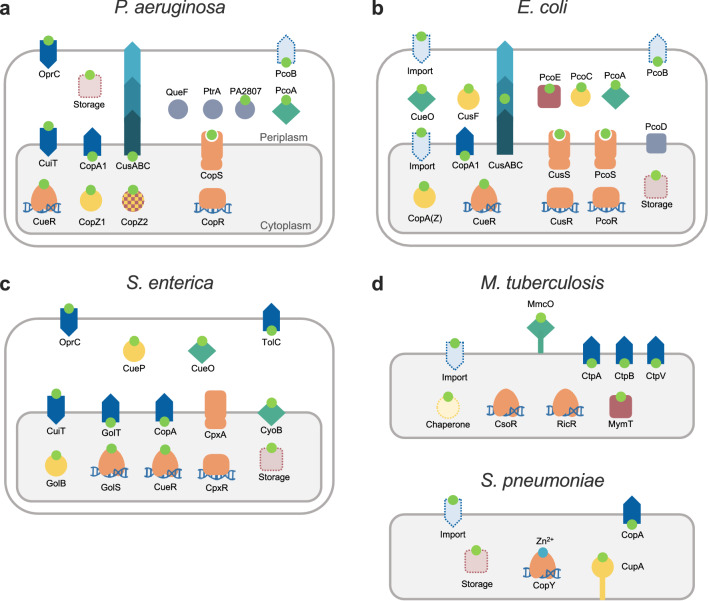
Alternative Cu^+^-homeostasis architectures in selected model pathogenic bacteria. Basic elements from the Cu^+^-homeostasis network depicted in Fig. 2 are identified in the Gram-negative species *P. aeruginosa* (**a**), *E. coli* (**b**), and *S. enterica* (**c**), and in the Gram-positive *M. tuberculosis* and *S. pneumoniae* (**d**)*.* Cu^+^ (lime circle), sensor (orange), storage (rose square), chaperone (yellow circle), transporters (blue arrow), cuproproteins (turquoise diamond). Only Cu^+^-regulated elements of the Cu^+^-distribution network are shown. Uncharacterized proteins whose expression depends on Cu^+^ levels are shown as gray circles. Shapes in dotted lines highlight missing elements from the proposed minimum model of Cu^+^ homeostasis

An initial analysis shows that there are well-conserved elements forming the common network cores in the Gram-negative species compared here (*P. aeruginosa, S. enterica,* and *E. coli*). These are the cytoplasmic transcriptional regulator CueR, CopZ-like chaperones, CopA-like exporters, and periplasmic TCS and MCOs (Fig. 4). However, differences emerge when the attention is directed to the regulation of these elements, alternative proteins performing similar roles, and homologous proteins fulfilling different functions. Moreover, there are necessary influx and efflux transporters still to be identified in some of these organisms. For instance, the *E. coli* CueR-regulon includes an MCO, a CopA1-like pump, and a CopZ-like chaperone (Fig. 4A) [35, 90, 92, 120]. Alternatively, the *P. aeruginosa* CueR regulator controls the expression of two Cu^+^ chaperones (CopZ1 and CopZ2), and two efflux systems (CopA1 and CusCBA), but the likely MCOs are regulated by CopSR (Fig. 4B) [24, 40]. Furthermore, the *S. enterica* CueR controls only CopA and the periplasmic MCO CueO, while the only apparent cytoplasmic chaperone GolB is regulated by GolS, a second CueR-like sensor (Fig. 4C) [121, 136]. GolS also regulates an additional CopA-like exporter GolT [121, 136]. The presence and function of the CusCBA system are interesting when exploring the alternative architectures that bacteria use to maintain Cu^+^ homeostasis, as it differs in the three Gram-negative species compared here. In *E. coli*, the CusCBA operon includes the periplasmic chaperone CusF and it is under the control of the periplasmic TCS sensor CusRS [126, 167, 179, 185]. Conversely, the *P. aeruginosa* CusCBA transporter is not part of the periplasmic CopRS regulon and is controlled by the cytoplasmic CueR [40]. Even more divergent, *S. enterica* lacks the entire *cusSRCFBA* locus as a result of its selective deletion during evolution and the concomitant acquisition of the *cueP* gene [136]. Other variations can be found in periplasmic Cu^2+/+^ sensing TCSs. While *E. coli* possesses two (CusRS and PcoRS), *P. aeruginosa* has only one (CopRS), and *S. enterica* apparently has none. However, it has been shown that *S. enterica* uses an alternative strategy to control the expression of the periplasmic chaperone CueP, based on the interplay of the cytoplasmic Cu^+^ sensor CueR and the general envelope stress response TCS CpxRA (also controlling the expression of the periplasmic thiol oxidoreductase Scs system) [34]. This arrangement reinforces the idea that compartmental Cu^+^ quotas are independently controlled, even if the regulation rests on sensing a secondary effect of Cu^+^ overload such as redox stress. Further highlighting the versatility of Cu^+^ sensing TCS, *E. coli* CusS functions via the kinase activity of the cytoplasmic phosphotransferase domain, while the *P. aeruginosa* CopS response relies on the phosphatase activity of its homologous cytoplasmic domain.

In contrast to the Gram-negative species, *S. pneumoniae* and *M. tuberculosis* depend on Cu^+^ sensing repressors (CsoR and CopY) to maintain the cytoplasmic Cu^+^ quota (Fig. 4D). *M. tuberculosis* has a quite distinct Cu^+^ homeostasis network. Perhaps not only due to the different environments it faces during infection, but also, because it manipulates, and evades the immune response, including the Cu^+^ overload used by the host immune system [199, 200]. *M. tuberculosis* encodes two Cu^+^ sensors (CsoR and RicR), three Cu^+^ ATPases (CtpA, CtpB, and CtpV), a Cu^+^ storage metallothionein (MymT), and the MCO Mmco that is released into the mycobacteria containing phagosome [98, 104, 168, 171, 188, 201–203]. Intriguingly, no Cu^+^ chaperone has been identified in this species. Also unknown is how Cu^+^ is channeled through the highly hydrophobic mycobacterial cell wall and enters the cytoplasm. *S. pneumoniae* CopY-controlled cytoplasmic Cu^+^ network includes the singular CupA chaperone (Fig. 4D) [123]. It is not clear how a membrane-anchored chaperone fulfills its role in Cu^+^ traffic, including Cu^+^ reception from a still unknown importer and transferring to and from CopY. Also unknown is whether Csp3, encoded in the *S. pneumoniae* genome, is a functional Cu^+^ storage system in vivo, or if other forms of storage system operate in this organism.

We propose that there are no redundant molecules in these networks, instead, each protein contributes to an independent Cu^+^ pool directed to separate targets and fulfilling different physiological roles. For example, in the case of the CopZ-like chaperones, while the low abundance CopZ1 provides Cu^+^ to CueR, the more abundant CopZ2 constitutes a cytoplasmic buffer/storage sink [24]. Likewise, CopA1 is responsible for cellular Cu^+^ detoxification and CopA2 provides the metal for periplasmic cuproenzyme metallation [42]. However, the assumed non-redundancy among apparently overlapping or complementing activities still needs further confirmation. For instance, the distinct roles of CopA and GolT or GolS and CueR in *S. enterica* that apparently work with a single chaperone GolB. These systems might just enable a distinct regulation but alternatively, distribute distinct Cu^+^ pools to different targets.

**Fig. 5:**
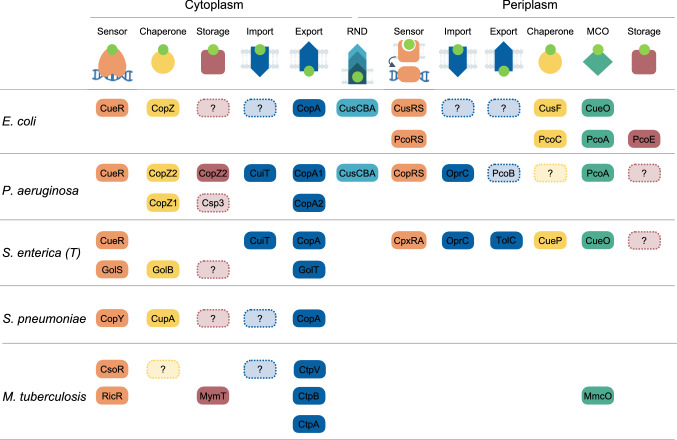
Parallelism of Cu^+^ network architectures in model bacteria. Top row shows the different elements from the Cu^+^ homeostatic network found in Gram-positive and Gram-negative bacteria. Subsequent rows identify each of those elements on the model bacteria compared here: *P. aeruginosa*, *E. coli*, *S. enterica*, *S. pneumoniae*, and *M. tuberculosis*. Shapes in dotted lines highlight missing elements from the proposed minimum model of Cu^+^ homeostasis

A parallel comparison of the Cu^+^ homeostasis elements described in the model bacteria points out the presence of core molecules, sensors, chaperones, and transporters involved in Cu^+^ distribution (Fig. 5). Furthermore, it also highlights missing elements (dotted lines) essential to interconnect the networks and maintain Cu^2+/+^ homeostasis in the absence of free Cu^+^. Among these, it is remarkable the limited available information on bacterial Cu^+^ importers and storage/sink molecules. It is also clear that bacteria have dedicated networks to maintain the Cu^+^ quota in each compartment. Thus, the multicompartmental Gram-negative bacteria use more elaborated/populated Cu^+^ mobilization systems to cross two impermeable membranes and move the metal through both, the cytoplasm, and periplasm, while the Gram-positive bacteria only need to transport the metal to and from the cytoplasm and mobilize it to proper targets to avoid its toxicity.

## Summary

The singular chemical characteristics of Cu^+^ have driven its use in several catalytic activities in bacterial cells. In parallel, these characteristics as well as those of biological systems (aqueous environment, impermeable membranes, abundance of reactive groups) have driven the evolution of singular distributions systems. Toward integration and aiming to provide a broad view of bacterial Cu^+^ distribution, system-wide conceptual and mathematical models have been proposed. The structure and biochemical characteristics of most of the molecular elements in these models have largely been established. This has enabled a significant understanding of Cu^+^ transfer and transport mechanism, and their regulation. The alternative strategies that emerged in bacteria to distribute the metal among the different compartments points to future lines of research by integrating the network components using system and omics approaches.
